# Temporal Dynamics and the Contribution of Plant Organs in a Phenotypically Diverse Population of High-Yielding Winter Wheat: Evaluating Concepts for Disentangling Yield Formation and Nitrogen Use Efficiency

**DOI:** 10.3389/fpls.2019.01295

**Published:** 2019-10-29

**Authors:** Lukas Prey, Yuncai Hu, Urs Schmidhalter

**Affiliations:** Chair of Plant Nutrition, Technical University of Munich, Munich, Germany

**Keywords:** yield physiology, breeding traits, yield prediction, early phenotyping, nitrogen translocation, phenomics, nitrogen allocation and partitioning, selection

## Abstract

Enhancing crop nitrogen use efficiency (NUE) is a key requirement for both economic and ecological reasons. Consequently, the genotypic potential for NUE in winter wheat (*Triticum aestivum L.)* requires further exploitation. Emerging plant phenomic techniques may provide knowledge about traits contributing to grain N uptake (GNup) and grain yield (GY). However, the understanding of beneficial strategies concerning the temporal dynamics of NUE and GY formation and the role of plant organs is still scarce especially under high-yielding European conditions—particularly to discriminate interesting lines in the breeding process. Thus, screening for potentially useful NUE traits in terms of variation, stability, and contribution to target traits will be an essential prerequisite for the development of efficient phenotyping strategies. Therefore, 46 NUE and yield formation traits were assessed in a population of 75 breeding lines over 3 years from 2015 to 2017 in southern Germany, including dry matter (DM), N concentration, and N uptake at anthesis and maturity, both at the aboveground-plant and plant organ levels. Significant genotype and genotype^x^environment effects were observed for all traits. While GY was more related to post-anthesis assimilation, also DM translocation contributed substantially to GY by 31–44%. At maturity, total aboveground DM as opposed to harvest index predominantly determined GY. NUE for GY was better described by N uptake efficiency than by N utilization efficiency. GNup was greatly influenced by variation in GY, but not in grain N concentration, and by total N uptake and not the N harvest index. Post-anthesis N uptake highly depended on the year and was low in comparison to N translocation. However, post-anthesis N uptake was always correlated with GNup, suggesting the need to also consider stay-green strategies under temperate growing conditions. While anthesis traits were only moderately descriptive, GY will be enhanced by increasing total biomass and the N uptake efficiency. Similarly, targeting total N uptake, particularly at post-anthesis, seems to be a rewarding strategy to boost GNup. Thus, high-throughput phenotyping should be targeted rather toward detecting traits related to DM and N acquisition than to the internal allocation and rather to post-anthesis than to anthesis traits.

## Introduction

For wheat, which contributes approximately 20% to the global calorific consumption (Reynolds et al., 2012), enhanced breeding efforts are required both to increase the grain yield (GY) to satisfy the growing demand (Zeigler and Mohanty, 2010; Grassini et al., 2013; Ray et al., 2013) and to increase the nitrogen use efficiency (NUE) for reducing the ecological impacts of the nitrogen (N) surplus (Erisman et al., 2008; Zhang et al., 2015). Globally, wheat removes less than half of the applied N through the harvest of produce (Zhang et al., 2015; Schlesinger, 2009; Galloway and Cowling, 2002; Lassaletta et al., 2014). While breeding significantly increased NUE (Garnett et al., 2015; Cormier et al., 2016; Lammerts van Bueren and Struik, 2017), promising advances in plant genotyping are still hampered by the scarce availability of field reference data (Furbank and Tester, 2011; Han et al., 2015;Araus et al., 2018). Recent advancements in plant phenotyping are promising for addressing this “phenotyping bottleneck” (Barmeier et al., 2017; Frels et al., 2018), but a better understanding of the NUE traits and how the factors interact is required (Nguyen and Kant 2018).

NUE relates the amount of overall or harvestable biomass to available or fertilized nitrogen. NUE can be dissected further into N uptake efficiency (NupEff), linking N uptake (Nup) to the available or fertilized amount of N, and N utilization efficiency (NutEff), which links the amount of overall or harvestable biomass with the Nup (Moll et al., 1982; Cassman et al., 2002; Foulkes et al., 2009; Equation 1 e + f; **Supplementary Equation 1**). Both NupEff and NutEff contributed to the NUE breeding progress (Cormier et al., 2013) but their influences differ based on the N conditions (Ortiz-Monasterio et al., 1997). The formation and the interactions among traits of NUE—including GY and grain N uptake—can be explored through various concepts (Hirel et al., 2007; Foulkes et al., 2009; Hawkesford, 2014; Fixen et al., 2015; Han et al., 2015; Equation 1). Grain Nup (GNup), the product of grain N concentration (GNC) and GY, determines the N removal rate of a cropping system (Equation 1 a), but GNC and GY are negatively correlated (Feil, 1997). The residuals from this relationship as expressed by the grain protein deviation (GPD) was suggested for a simultaneous optimization of GY and GNC (Monaghan et al., 2001; Oury and Godin, 2007). While GPD may hold the advantage of being more heritable (Thorwarth et al., 2018), and selecting for high GPD would maximize GNC for a given GY level, rather genotypes high in GY were found to be superior in GNup also (Rapp et al., 2018).

In the face of stricter fertilizing legislations and discussions about the sufficient level of GNC (Gabriel et al., 2017), the maximization of GNup or protein yield is gaining more attention (Koekemoer et al., 1999; Rapp et al., 2018; Michel et al., 2019).

**Equation 1:** Concepts applied to dissect NUE into grain yield (GY), grain N uptake (GNup) and grain N concentration (GNC): GNup as the product of GY and GNC (a), GY as the product of the yield components spike density (SD), grain number per spike (GNS) and thousand kernel weight (TKW; b), GY as the sum of dry matter translocation (DMT) and post-anthesis assimilation (PAA; c), GY as the product of total DM and harvest index (HI; d), NUE as product of N uptake efficiency and N utilization efficiency with respect to NUE for total DM production (e) and NUE for GY, respectively (f), GNup as the sum of N translocation (NT) and post-anthesis N uptake (PANup; g) and GNup as the product of N uptake (Nup) and the N harvest index (NHI; h). See **Supplementary Equation 1** for an extended versions of (f) in relation to (a), (d), (e) and (h).

(1) $$\begin{array}{l} (a)\;\mathrm{GNup}=\mathrm{GY}*\mathrm{GNC}\\ (b)\;\mathrm{GY}=\mathrm{SD}*\mathrm{GNS}*\mathrm{TKW}\\ (c)\;\mathrm{GY}=\mathrm{DMT}+\mathrm{PAA}\\ (d)\;\mathrm{GY}\;\mathrm{DM}*\mathrm{HI}\\ (e)\;{\mathrm{NUE}}_{\mathrm{total}}\;\mathrm{NupEff}*{\mathrm{NutEff}}_{\mathrm{total}}\\ (f)\;{\mathrm{NUE}}_{\mathrm{grain}}\;\mathrm{NupEff}*{\mathrm{NutEff}}_{\mathrm{grain}}\\ (g)\;\mathrm{GNup}=\mathrm{NT}+\mathrm{PANup}\\ (h)\;\mathrm{GNup}=\mathrm{Nup}*\mathrm{NHI} \end{array}$$

Yield formation in wheat is highly compensatory and can be rarely ascribed to one of the components spike density (SD), grains per spike (GNS), or thousand kernel weight (TKW; Equation 1 b; Cormier et al., 2013). Increased GNS at rather constant TKW (Hay 1995) or increased spike and grain numbers per m², but not increased GNS or TKW (Shearman et al., 2005), were observed for high-yielding genotypes. From a temporal perspective, GY formation can be dissected into pre-anthesis contribution, i.e., the translocation of DM (DMT) and post-anthesis assimilation (PAA; Equation 1 c). The contribution of PAA to grain filling (CPAA) has often been reported to be well above 50% (Savin and Slafer, 1991). In contrast, the contribution of DMT to GY approached 60% under heat stress (Plaut et al., 2004) and 100% under drought stress (Van Herwaarden et al., 1998; Inoue et al., 2004). Grain yield can be limited by sink size (Borrás et al., 2004; Fischer, 2007; Foulkes et al., 2011; Slafer et al., 2014), thus that it could be indirectly predicted based on canopy traits during anthesis (Demotes-Mainard and Jeuffroy, 2004; Foulkes et al., 2009). In contrast to the green revolution (Hay, 1995; Sinclair 1998), in recent decades, yield gains are more attributed to increased total DM than to better relative partitioning of assimilates to the grain as expressed by the harvest index (HI; Equation 1 d; Shearman et al., 2005; Cormier et al., 2013).

Unlike for GY, the major component (60–95%; Hirel et al., 2007) of GNup is N remobilized and translocated during the grain filling phase (N translocation, NT) as opposed to post-anthesis Nup (PANup; Equation 1 g; Masclaux-Daubresse et al., 2010; Barraclough et al., 2014; Kong et al., 2016). NT—influenced by pre-anthesis Nup and the efficiency of its translocation (NTEff; Barbottin et al., 2005)—is associated with senescence and, therefore, could counteract yield formation (Foulkes et al., 2009; Masclaux-Daubresse et al., 2010; Gaju et al., 2011). Culm and spike N, which are translocated during grain filling, act as reserve pools and delay the remobilization of leaf N (Foulkes et al., 2009; Pask et al., 2012). The NTeff reached 43 to 92% depending on the plant organ, the environment, and disease pressure (Barbottin et al., 2005; Foulkes et al., 2009; Kong et al., 2016). In contrast to carbon accumulation, NT and post-anthesis N uptake are considered mostly source-limited (Martre et al., 2003; Bancal 2009; Foulkes et al., 2009; Masclaux-Daubresse et al., 2010). In spite of the lower contribution compared to NT, post-anthesis N uptake seems to be a key driver of variation in GNup (Cox et al., 1985; Bogard et al., 2011).

Final GNup is the product of total Nup and N harvest index (NHI; Equation 1 h; Sinclair 1998), which was reported to be closely correlated to NT efficiency (Hirel et al., 2007; Fageria 2014). NHI can vary substantially (Guttieri et al., 2017), but it remains unclear if it is increased through higher NT efficiency or post-anthesis N uptake (Cormier et al., 2013).

In spite of numerous studies having assessed variation in N and DM allocations, most of the conclusions were drawn from selected cultivars, often including varying N fertilization treatments or a wide range of historical cultivars to assess the effects of breeding. Moreover, in most cases, only few NUE and GY concepts were addressed, hindering the comparison of their use across studies. In face of the ongoing breeding process, a comprehensive assessment of the various concepts on current genotypes is missing. Thus, relatively little is known about the relationships among traits and the potential use of indirect traits for early in-season estimation of target traits, such as grain yield, grain N uptake, and GNC in breeding lines, particularly under temperate high-yielding West European conditions, and with respect to the allocation to plant organs. Therefore, the aim of the present study was (i) to assess the variation and stability in various aboveground organ- and plant-level dry matter and N traits at anthesis and maturity as well as (ii) their relationships with the target NUE traits (Equation 1).

## Materials and Methods

### Study Site and Experimental Design

The field experiment was conducted over 3 years from 2014/2015 to 2016/2017 for evaluating traits that influence NUE and yield formation in a diverse population of winter wheat double haploid breeding lines. The population’s parents consisted of elite cultivars and breeding lines provided by regional plant breeders. The population had undergone pre-selection, which removed genotypes peculiar in terms of extreme flowering date, plant height, and disease susceptibility. In the first year, the trial comprised the complete population of 400 genotypes, which were reduced to a random subset for the further sampling. Thus, the study comprised 75 lines in 2014/2015 in two replicates, 75 lines in 4 replicates in 2015/2016, and 32 selected lines representing the overall yield variation in 4 replicates in 2016/2017. In addition, three high-performance cultivars (*“JB Asano,” “Elixer,” “Julius”*) were included as references. Plot width was 1.5 m and plot length 6.5 m. The trial site was at the Dürnast field research station of the Technical University of Munich (48.406 N, 11.692 E). The soil was mainly composed of homogenous Cambisols of loamy clay. The preceding crops were wheat in the first and second year and grass-clover in the third year. The trials were sown in October–November (**Table 1**). Seeds were fungicide-dressed, and leaf fungicide was sprayed in three applications in 2015 and in two applications in 2016 and 2017. Chlormequat-based straw shortener was used to prevent lodging in all years. According to local practices, N fertilization was split into three dressings with the highest amount applied at the beginning of vegetation in spring followed by tillering and booting-anthesis (Table 1). N fertilizer summed up to 200 kg N ha^–1^ in the first and second years, but only 130 kg N ha^–1^ in the third year, following the consideration of high N mineralization rates from the preceding grass–clover. Fertilizer was applied as combined ammonium/nitrate granules. An N fertilization experiment including a zero N treatment was conducted in direct proximity to the experiment in all years and served as a proxy for the influence of soil N supply on yield and N yield (Table 1). The soil N supply was low in the first year, resulting into only 20 kg N ha^–1^ N yield without fertilization compared to 44 kg N ha^–1^ and 111 kg N ha^–1^ in 2016 and 2017, respectively.

**Table 1:** Crop management data in the main experiment, as well as yield and N-yield in the non-fertilized plots (N0) in the three experimental years. N_fert_ I, II, and III indicate amounts and application dates for N dressings. For sowing, fungicide, and growth regulator, application dates (month/day) are given.

Year	Sowing density (kernels m^–2^)	Sowing date	Fungicide	Growth regulator	N_fert_ I	N_fert_ II	N_fert_ III	Grain yield N0 (n = 8)	Grain N-yield N0 (n = 8)
2014/15	350	11/04	05/12; 05/27; 06/05	04/21	03/19 (80 kg)	05/11 (60 kg)	06/11 (60 kg)	15 dt ha^-1^	20 kg ha^-1^
2015/16	350	10/13	04/22; 06/07	04/04	03/22 (80 kg)	04/29 (70 kg)	05/23 (50 kg)	37 dt ha^-1^	44 kg ha^-1^
2016/17	350	10/24	05/18; 05/30	04/11; 05/18	03/27 (50 kg)	05/18 (50 kg)	06/08 (30 kg)	70 dt ha^-1^	111 kg ha^-1^

**Figure 1:**
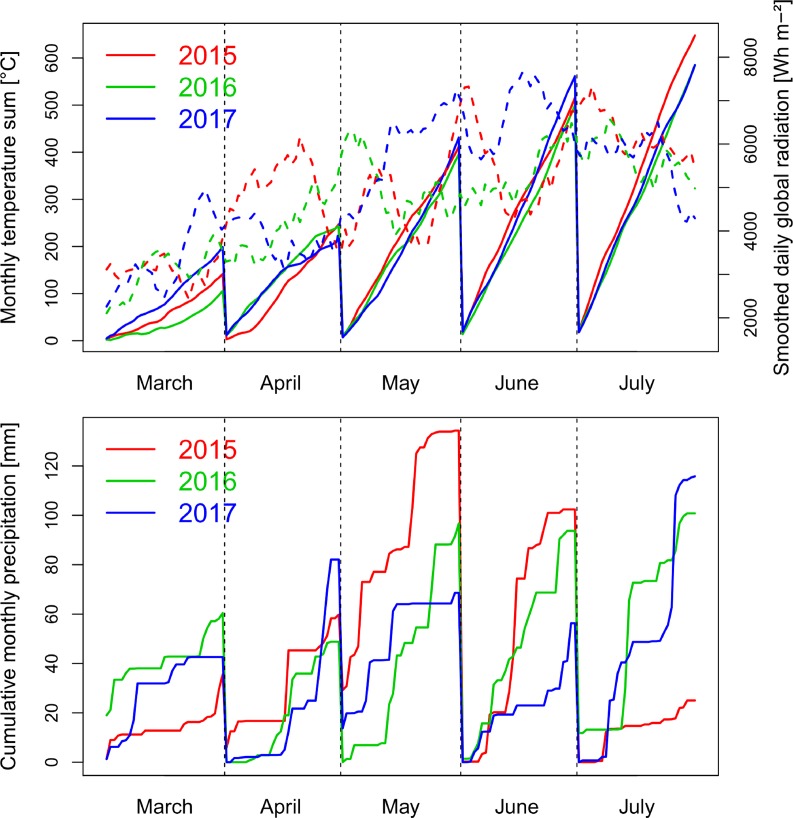
Weather conditions with global radiation smoothed by a 10 day-moving average (dashed lines), monthly cumulative temperature and cumulative precipitation during the three growing seasons from October to July. In all years, anthesis took place in the first half of June.

Precipitation in the main wheat growing period from October to August was 714 mm in 2014/15, 746 mm in 2015/16 and 690 nm in 2016/17. The first growing season was characterized by a wet May in 2015 and low global radiation, followed by warm and dry conditions during July (**Figure 1**). The conditions caused visible heat and drought effects resulting in accelerated senescence.

In contrast, the conditions were moderate in spring 2016 with lower levels of radiation during grain filling in June and July, more equally distributed precipitation, and lower temperatures from May until July. Due to better sowing conditions, suitable growing conditions, and the warm April, vegetative growth was higher in 2016, resulting into visible pathogen pressure, being further influenced by the only two fungicide applications. The year 2017 was characterized by increased radiation during May and June, in addition to reduced but overall sufficient precipitation, and higher June temperatures than in the previous years.

### Plant Sampling and Plant Analysis

Biomass sampling was conducted at mid-flowering (Zadok’s growth stage 65; Zadoks et al., 1974) and at physiological maturity (stage 95). At flowering, sampling dates were determined for each genotype by visual scoring. For the anthesis sampling date, 20 randomly selected spiked culms were cut directly at the culm base in 2015 and 30 culms in 2016 and 2017. At maturity, 30 culms were removed in 2015 and 50 culms in 2016 and 2017. The plants were manually separated into leaves, culms including leaf sheaths, and spikes. At maturity, spikes were threshed into grain and chaff. In 2016, among the 78 sampled genotypes, only samples of 34 randomly selected genotypes were separated by plant organs while the rest of the genotypes were analyzed as aboveground plants at anthesis and threshed into straw/grain at maturity. Plant samples were oven-dried at 50°C until a constant weight was attained for the subsequent determination of dry weight. The vegetative plant parts were milled using a 1 mm sieve for detecting N concentration (NC) using near-infrared spectroscopy (NIRS), using a FOSS NIRS 6500 (NIRSystem, Silver Spring, Md., US) and a Fourier transform NIRS (Bruker, MPA, Billerica, Mass., US). Grains were analyzed as complete kernels. After sampling the plants at maturity, all plants in the plots were harvested using a combine harvester, and grain yield of each plot was determined. In addition, TKW was determined for each plot. The grain numbers in the manually harvested shoots were determined to estimate the number of GNS. By incorporating the information of yield per spike and plot yield, the spike density per m² (SD) was calculated. Nitrogen uptake (Nup) was calculated by multiplying NC with DM. Plant height was determined during milk ripeness using an ultrasonic triangulation sensor (Barmeier et al., 2016). Anthesis dates were recorded as days in June when the plants were at mid-flowering.

### Assessment of Derived Plant Traits

Indirect traits were assessed based on yield components, including DM and NT, and N uptake and utilization efficiency (**Table 2**; for full names of abbreviations see **Supplementary Table 1**). DM values of all plant components corresponding to the number of sampled shoots were scaled up to kg ha^−1^ using the spikes per m² values for each plot. To facilitate comparisons between cultivars based on translocation processes of assimilates and nitrogen, the following parameters were assessed: absolute amount of pre-anthesis accumulated assimilates translocated from vegetative plant organs into grains between anthesis and maturity in kg ha^−1^: dry matter translocation (DMT; Papakosta and Gagianas, 1991):

$$\begin{array}{c} \mathrm{DMT}=\mathrm{DM}{\left(\mathrm{spikes}+\mathrm{stems}+\mathrm{leaves}\right)}_{\mathrm{anthesis}}\\ –\mathrm{DM}{\left(\mathrm{chaff}+\mathrm{stems}+\mathrm{leaves}\right)}_{\mathrm{maturity}} \end{array}$$

**Table 2:** Heritability estimates and descriptive mean values for all DM and N-traits from the three experimental years. Heritability values were colored from low (red) to high (blue) values. See **Supplementary Table 1** for a description of all traits and **Supplementary Table 2** for ANOVA results and further descriptive measures. If not indicated, traits are unitless.

Trait group	Trait	Heritability			Mean		
		2015	2016	2017	2015	2016	2017
**DM traits (kg ha**^−^**^1^)**	**Total Ant**	0.51	0.53	0.73	8,245	13,303	12,085
	**Leaves Ant**	0.59	0.73	0.84	1,430	2,381	2,638
	**Culms Ant**	0.53	0.69	0.78	5,211	8,166	6,819
	**Spikes Ant**	0.61	0.65	0.78	1,604	2,330	2,627
	**Total Mat**	0.52	0.77	0.85	12,544	18,005	18,948
	**Leaves Mat**	0.59	0.92	0.84	932	1,292	1,575
	**Culms Mat**	0.63	0.92	0.85	3,539	6,066	5,168
	**Chaff Mat**	0.39	0.86	0.85	1,386	2,102	2,297
	**Grain Mat (GY)**	0.60	0.87	0.88	6,687	8,380	9,908
**N concentrationtraits** (%)	**NC leaves Ant**	0.49	0.78	0.82	2.72	3.00	3.51
	**NC culms Ant**	0.58	0.90	0.78	0.82	1.04	1.17
	**NC spikes Ant**	0.66	0.92	0.89	1.70	1.75	1.91
	**NC leaves Mat**	0.87	0.96	0.80	0.93	1.20	0.80
	**NC culms Mat**	0.87	0.72	0.73	0.29	0.34	0.45
	**NC chaff Mat**	0.58	0.87	0.76	0.56	0.53	0.58
	**NC grain Mat (GNC)**	0.78	0.88	0.87	2.20	1.96	2.32
**N uptake traits (kg ha^−1^)**	**Total Ant**	0.33	0.58	0.61	109	207	223
	**Leaves Ant**	0.53	0.73	0.75	39	72	92
	**Culms Ant**	0.30	0.72	0.64	42	85	80
	**Spikes Ant**	0.55	0.68	0.90	27	41	50
	**Total Mat**	0.11	0.64	0.81	173	213	278
	**Leaves Mat**	0.58	0.89	0.77	9	15	13
	**Culms Mat**	0.42	0.77	0.79	10	21	23
	**Chaff Mat**	0.16	0.83	0.83	8	11	13
	**Straw Mat**	0.08	0.69	0.79	27	49	49
	**Grain Mat (GNup)**	0.34	0.76	0.82	147	164	229
**Derived DM traits + yield components**	**DMTEff**	0.48	0.66	0.59	0.29	0.27	0.25
	**PAA (kg ha^–1^)**	0.63	0.67	0.70	4,302	4,693	6,864
	**DMT (kg ha^–1^)**	0.49	0.59	0.52	2,385	3,701	3,045
	**HI**	0.65	0.83	0.89	0.53	0.47	0.52
	**TKW (g)**	0.93	0.94	0.97	39	35	37
	**Spike density (m^–2^)**	0.77	0.82	0.95	326	524	657
	**GNS**	0.88	0.87	0.96	54	46	41
	**CPAA**	0.56	0.66	0.57	0.64	0.56	0.69
	**NutEff_total**	0.67	0.78	0.77	73	85	68
	**NutEff_grain**	0.80	0.83	0.83	39	39	36
	**NUE_total**	0.52	0.77	0.85	63	90	146
	**NUE_grain**	0.60	0.87	0.88	33	42	76
**Derived N traits**	**NTEff**	0.70	0.75	0.63	0.75	0.76	0.78
	**PANup (kg ha^–1^)**	0.49	0.75	0.23	65	5	56
	**NT (kg ha^–1^)**	0.55	0.62	0.42	82	159	173
	**NT leaves (kg ha^–1^)**	0.61	0.78	0.74	30	56	80
	**NT culms (kg ha^–1^)**	0.45	0.80	0.50	32	64	57
	**NT spikes (kg ha^–1^)**	0.63	0.65	0.78	19	29	37
	**NHI**	0.66	0.86	0.81	0.85	0.77	0.82
	**CPNup**	0.60	0.72	0.16	0.37	0.02	0.20
**Other traits**	**Plant height (m)**	0.87	0.95	0.85	0.61	0.93	0.65
	**Days to anthesis (d)**	–	–	–	12.85	10.61	12.87

Ant, anthesis; CPAA, contribution of post-anthesis assimilation to grain-filling; CPNup, contribution of post-anthesis nitrogen uptake to total nitrogen uptake; DM, dry matter; DMT, dry matter translocation; DMTEff, dry matter translocation efficiency; HI, harvest index; GNC, grain nitrogen concentration; GNS, grain number per spike; GNup, grain nitrogen uptake; GY, grain yield; Mat, maturity; N, nitrogen; NC, nitrogen concentration; NHI, nitrogen harvest index; NT, nitrogen translocation; NTEff, N translocation efficiency; NUE, N use efficiency; NutEff, N utilization efficiency; PAA, post-anthesis assimilation; PANup, post-anthesis nitrogen uptake; TKW, thousand kernel weight. Number of genotypes: 2015: n = 78; 2016: n = 78 and n = 34 for traits related to leaves, chaff/spikes and stems; 2017: n = 35.

Relative amount of pre-anthesis accumulated assimilates translocated into grains (Papakosta and Gagianas, 1991): DMT efficiency (DMTEff):

$$\mathrm{DMTEff}=\mathrm{DMT}/{\mathrm{DM}}_{\mathrm{anthesis}}$$

Post-anthesis assimilation (PAA):

$$\mathrm{PAA}={\mathrm{DM}}_{\mathrm{maturity}}–{\mathrm{DM}}_{\mathrm{anthesis}}$$

Contribution of post-anthesis assimilates to grain filling (CPAA):

$$\mathrm{CPAA}=\mathrm{PAA}/{\mathrm{DM}(\mathrm{grain})}_{\mathrm{maturity}}$$

Ratio of grain DM to total DM at maturity (harvest index, HI):

$$\mathrm{HI}={\mathrm{DM}}_{\mathrm{grain}}/{\mathrm{DM}}_{\mathrm{total}}$$

Absolute amount of pre-anthesis accumulated nitrogen translocated from vegetative plant organs into grains between anthesis and maturity in kg ha^−1^ (NT; Cox et al., 1985):

$$\mathrm{NT}=\mathrm{Nup}\;{\left(\mathrm{spikes}+\mathrm{stems}+\mathrm{leaves}\right)}_{\mathrm{anthesis}}-\mathrm{Nup}{\left(\mathrm{chaff}+\mathrm{stems}+\mathrm{leaves}\right)}_{\mathrm{maturity}}$$

Accordingly, partial NT was calculated for spikes, culms, and leaves.

Relative amounts of pre-anthesis accumulated nitrogen translocated into grains (Cox et al., 1985): NT efficiency (NTEff):

$$\mathrm{NTEff}=\mathrm{NT}/{\mathrm{Nup}}_{\mathrm{anthesis}}$$

Post-anthesis nitrogen uptake (PANup):

$$\mathrm{PANup}={\mathrm{Nup}}_{\mathrm{maturity}}–{\mathrm{Nup}}_{\mathrm{anthesis}}$$

Contribution of post-anthesis nitrogen to total nitrogen uptake (CPNup):

$$\mathrm{CPNup}=\mathrm{PANup}/{\mathrm{Nup}}_{\mathrm{maturity}}$$

Ratio of grain nitrogen uptake to total Nup at maturity (NHI):

$$\mathrm{NHI}={\mathrm{Nup}}_{\mathrm{grain}}/{\mathrm{Nup}}_{\mathrm{total}}$$

Efficiency of the internal conversion of N into total DM (nitrogen utilization efficiency, NutEff; Moll et al., 1982), where NutEff_total was calculated at anthesis and maturity:

$${\mathrm{NutEff}}_{\mathrm{grain}}={\mathrm{DM}}_{\mathrm{grain}}/{\mathrm{Nup}}_{\mathrm{total}}$$

$${\mathrm{NutEff}}_{\mathrm{total}}={\mathrm{DM}}_{\mathrm{total}}/{\mathrm{Nup}}_{\mathrm{total}}$$

Since only one N-fertilization level was considered in each year, total Nup was a direct function of N uptake efficiency, total DM of total NUE, and grain DM of NUE for grain (Moll et al., 1982), so that we did not include NupEff and NUE explicitly in the correlation analysis but indirectly through traits that are directly determinable, i.e., Nup and DM.

### Statistical Analysis

Statistical analyses were conducted in R 3.3.4 (R Stat. Core Team, 2017). All trait observations *t* were subjected to analysis of variance (ANOVA) across years based on the model t_ijkab_ = µ + y_i_ + g_j_ + (gy)_[ij]_ + b_[ik]_ + r_[ika]_ + c_[ikb]_+ ε_[ijkab]_. µ denotes the overall mean, *g* the effect of the j^th^-genotype in the b^th^-column (c) and the a^th^ row (r) within the k^th^ block (b) within the i^th^ year (y), and ε the error term, assuming fixed effects for *g* and *y* and random effects for the other factors. Type III-F-test was calculated using Satterthwaite’s approximation for the fixed effects. Heritability (H²) was estimated within years (H²) to assess the repeatability of the trait assessment setting all factors to random as H² = V_g_/(V_g_ + V_ε_/n_b_) (Holland et al., 2003). *V* denotes the variance components, and *n_b_* number of blocks. Correlations among the plant traits were calculated within the 3 years, using trait values averaged across replicates by genotypes, and compared using Pearson’s correlation coefficient, focusing on the relationships with GY, GNup, and GNC as main target traits. In addition, GPD was calculated as the residuals from the regression between GNC and GY (Monaghan et al., 2001). Selected interacting trait relationships were visualized in scatterplots with isolines (Oury and Godin, 2007; Bogard et al., 2010).

## Results

### Descriptive Statistics on Dry Matter and Nitrogen Traits

Significant genotype, year, and interaction effects were observed for almost all plant traits (**Supplementary Table 2**).

#### Components of Grain Yield Formation

**Figure 2:**
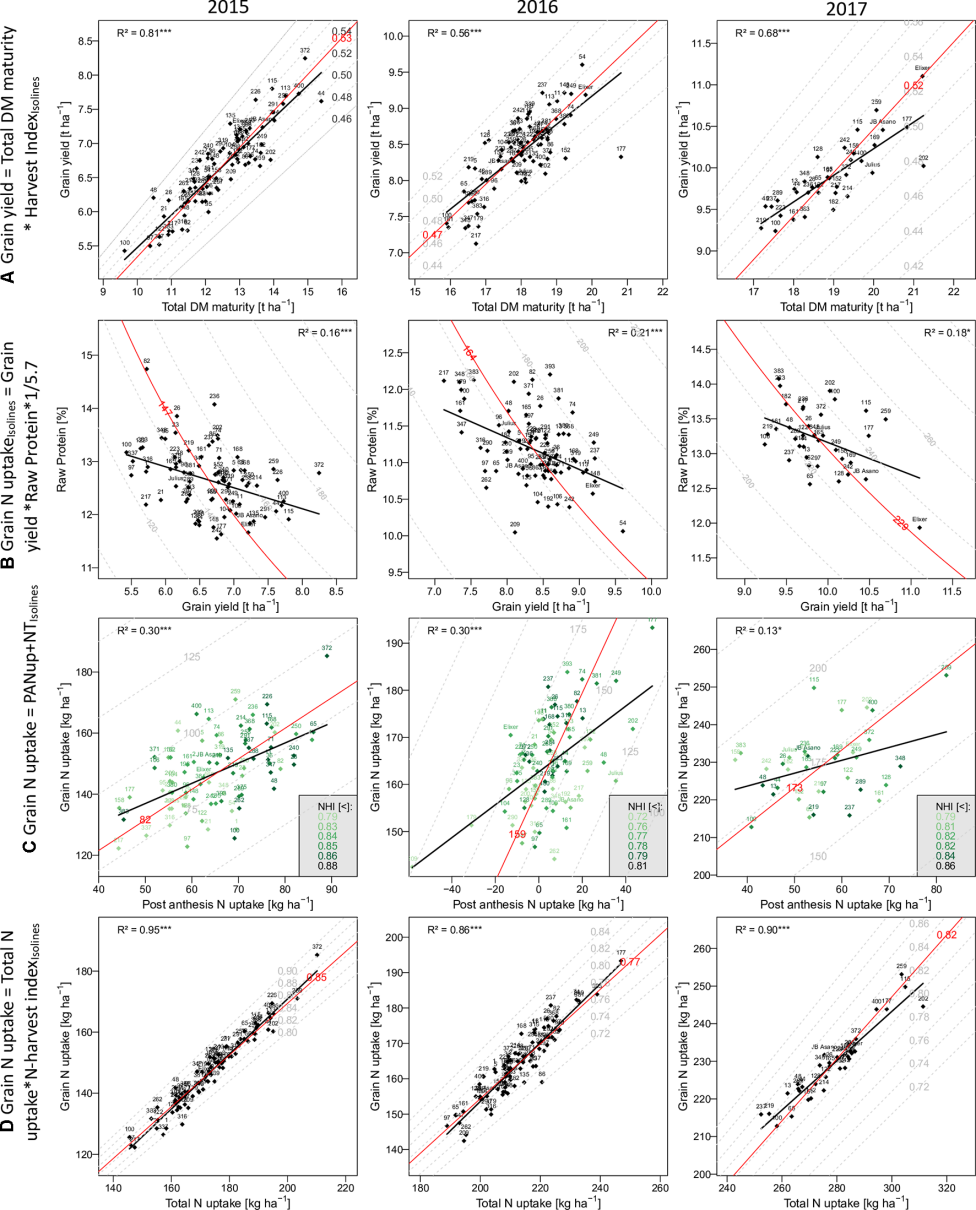
The relationship of selected traits with grain yield and grain N uptake: Traits on the y-axes are described as product**(A** and **D)** or as sum **(C)** of the traits on the x-axes and the dashed isolines. In **(B)** the grain N uptake on the isolines is the product of the x- and y-axes traits. Numbers and names indicate different breeding lines and the three reference cultivars, respectively. Red isolines and numbers indicate the position and average value of the isoline trait, respectively. Black regression lines and the coefficients of determination describe the relationship between the x- and y-axes traits.

Grain yield [DM grain at maturity (Mat)] increased over the years from 6.7 to 8.3 t ha^–1^, to 9.9 t ha^–1^ on average in the first, second, and third years, respectively (**Table 2**). High total DM at maturity (DM Mat) in 2016 was associated with a low HI (on average across genotypes 0.53, 0.47, and 0.52 in the 3 years; **Figure 2A**). Pre-anthesis assimilation was highest in 2016, with anthesis (Ant) DM summing up to on average of 13.3 t ha^−1^. In spite of slightly lower apparent DM translocation efficiency in 2016 (on average 27%) in comparison to 2015 (29 %), high Ant DM resulted into high DM translocation from vegetative organs into the grain in 2016 (3.7 t ha^–1^). In contrast, the PAA was similar in 2015 and 2016 (4.3 and 4.7 t ha^–1^ on average, respectively) but markedly increased in 2017 to 6.9 t ha^–1^. Consequently, it contributed on average 69% to grain yield (CPAA) in 2017, but only 64% and 56% in 2015 and 2016, respectively. Increasing grain yield over the years was associated with a doubling of the SD from only 326 spikes m^–2^ on average in 2015 to 657 spikes m^–2^ in 2017. In contrast, the grain number per spike decreased, and the TKW was comparable in 2015 and 2017 but lowest (35 g) in 2016.

#### Components of Grain Nitrogen Uptake

In comparison to grain yield, grain N uptake (GNup) only slightly increased from 2015 to 2016, from 147 kg N ha^–1^ on average to 164 kg N ha^–1^, due to lower GNC (on average 2.20 and 1.96%, respectively; **Table 2**; **Figure 2B**). In 2017, however, increases in both grain DM and GNC (2.32%) resulted in a significantly enhanced GNup of 229 kg N ha^−1^ on average and a maximum of 253 kg N ha^−1^ (**Figure 2B**). Similar to DM formation, Nup shifted toward the vegetative phase in 2016. Therefore, PANup was only 5 kg N ha^–1^ on average but differed substantially from a calculated 58 kg N ha^−1^ loss to a 52 kg N ha^–1^ increase in the individual lines (**Figure 2C**; **Supplementary Table 2**). In contrast, PANup summed up to 65 and 56 kg N ha^–1^ on average in 2015 and 2017, respectively. In turn, the apparent translocation of N (NT) from vegetative organs into the grain was the major source of GNup with 82, 159, and 173 kg N ha^–1^ in the 3 years, respectively (**Figure 2C**). Spike NT contributed only about 20% across all years, whereas culm NT contributed a fraction almost similar to that of leaf NT in 2015 (38%), but relatively more (43%) in 2016 and less (32%) in 2017. Among the plant organs, NT efficiency (NTEff) was always highest in leaves (0.78–0.86) and mostly lowest in spikes (0.71–0.73; not shown). The proportions of N allocated to spikes or chaff were fairly stable over the years and mostly constituted the smallest N pool at both Ant (on average 21 to 23%) and maturity (4–5%; not shown). The grain NHI was highest in 2015 (85%) and lowest in 2016 (78%; **Figure 2D**).

### Estimated Heritability

Heritability (H²) was generally higher in 2016 and 2017 than in 2015 (**Table 2**). With few exceptions, H² of “direct” DM and Nup was higher at maturity (Mat) than at Ant. In contrast, no consistent difference was found between vegetative organs. High H² values (H² > 0.72) were observed for N concentration traits in 2016 and 2017 and for GNC in all years. Similarly, all Nup traits were moderately heritable (>0.58) in 2016 and 2017 in contrast to low values in 2015. Both grain yield and GNup tended to be more heritable than total DM and total Nup, respectively. GNup yielded lower H² values than GY and GNC, similarly as observed for vegetative N uptake traits compared to corresponding DM and NC traits of the same organs. The three yield components together with the plant height (all H² > 0.83) were most heritable. Among the “derived” DM traits, HI (0.65–0.89), NutEff_total, and NutEff_grain were more heritable than traits related to DM translocation and PAA. Among the “derived” N traits, the NHI, NT of spikes and leaves, and the NT efficiency yielded H² values higher than 0.50 in all years.

### Early In-Season Correlations With Final Target Traits

From a phenotyping, predictive perspective, early estimation of the major target traits including GY, GNC, and GNup out of Ant traits is desirable. In all years, both total and culm DM, and total Nup at Ant were weakly to moderately correlated (*p* < 0.05) with GY (r = 0.43–0.57; **Table 3**). Correlations with leaf and spike DM were significant for GY only in 2015 and 2016. Organ-level Nup was descriptive (r = 0.55 for leaf Nup) mainly in 2015. Ant N concentration, flowering date, and plant height were not correlated with GY within the 3 years. In the case of GNup, correlations were observed with total Nup (r = 0.59 and 0.64) and DM at Ant, as well as with organ-level Ant Nup (highest for culm Nup) in 2015 and 2017. Among N concentration traits at Ant, only few correlations were observed and never in more than 1 year.

**Table 3:** Correlations (only p < 0.05; ***: p < 0.001; **: p < 0.01; *: p < 0.05) with target traits grain yield (GY), grain N concentration (GNC), and grain N uptake (Nup), by traits grouped as in Table 2. Bold numbers highlight correlations among the three target traits. Grey shades highlight correlations for traits potentially available already at anthesis. See **Supplementary Table 1** for a description of all traits.

	Grain yield			Grain N concentration			Grain N uptake		
	2015	2016	2017	2015	2016	2017	2015	2016	2017
Total DM Ant	0.57 ***	0.35 **	0.43 *		−0.23 *		0.56 ***		0.61 ***
DM leaves Ant	0.56 ***	0.51 **		−0.27 *			0.46 ***	0.44 **	0.50 **
DM culms Ant	0.57 ***	0.35 *	0.4 *				0.57 ***		0.58 ***
DM spikes Ant	0.36 **	0.46 **					0.4 ***	0.38 *	
Total DM Mat	0.90 ***	0.75 ***	0.82 ***	−0.30 **			0.81 ***	0.74 ***	0.69 ***
DM leaves Mat	0.62 ***	0.42 *	0.45 **	−0.25 *			0.53 ***	0.54 ***	0.66 ***
DM culms Mat	0.65 ***		0.56 ***				0.64 ***	0.45 **	0.55 ***
DM chaff Mat	0.37 ***	0.35 *	0.47 **				0.33 **	0.65 ***	
DM grain Mat (GY)	1.00***	1.00***	1.00***	−0.40 ***	−0.45 ***	−0.43 *	0.86 ***	0.66 ***	0.64 ***
NC leaves Ant							0.23 *		
NC culms Ant									
NC spikes Ant						0.44 **			
NC leaves Mat	−0.5 ***	−0.43 *		0.29 *			−0.38 ***	−0.40 *	
NC culms Mat	−0.52 ***			0.36 **	0.42 *		−0.36 **		
NC chaff Mat	−0.29 *				0.45 **	0.37 *	−0.25 *		0.4 *
NC grain Mat (GNC)	−0.40 ***	−0.45 ***	−0.43 *	1.00***	1.00***	1.00***		0.28 *	0.42 *
Total Nup Ant	0.60 ***	0.31 **	0.34 *			0.36 *	0.59 ***		0.64 ***
Nup leaves Ant	0.55 ***	0.43 *					0.48 ***		0.43 **
Nup culms Ant	0.56 ***					0.38 *	0.59 ***		0.57 ***
Nup spikes Ant	0.41 ***						0.46 ***		0.37 *
Total Nup Mat	0.82 ***	0.57 ***	0.61 ***		0.27 *	0.40 *	0.97 ***	0.93 ***	0.95 ***
Nup leaves Mat						0.37 *			0.61 ***
Nup culms Mat				0.23 *	0.40 *		0.23 *	0.35 *	0.48 **
Nup chaff Mat			0.38 *		0.39 *			0.34 *	0.50 **
Nup straw Mat			0.39 *						0.62 ***
Nup grain Mat (GNup)	0.86 ***	0.66 ***	0.64 ***		0.28 *	0.42 *	1.00***	1.00***	1.00***
DMTEff			−0.39 *					−0.28 *	
PAA	0.71 ***	0.42 ***	0.69 ***	−0.36 **		−0.45 **	0.57 ***	0.56 ***	
DMT					−0.25 *		0.23 *		
HI		0.56 ***			−0.42 ***			0.24 *	−0.37 *
TKW		0.36 **					0.28 *	0.43 ***	
Spike density	0.60 ***						0.59 ***	0.25 *	
GNS				−0.26 *	−0.25 *		−0.26 *		
CPAA	0.23 *		0.38 *			−0.37 *		0.41 ***	
NutEff_total	0.39 ***	0.29 **	0.46 **	−0.78 ***	−0.74 ***	−0.79 ***		−0.24 *	
NutEff_grain	0.53 ***	0.59 ***		−0.92 ***	−0.81 ***	−0.92 ***			−0.48 **
NTEff	0.52 ***	0.28 *					0.44 ***		−0.36 *
PANup	0.34 **		0.34 *	0.32 **	0.38 ***		0.54 ***	0.54 ***	0.36 *
NT	0.65 ***	0.34 **			−0.24 *	0.34 *	0.62 ***		0.53 **
NT leaves	0.58 ***	0.45 **		−0.24 *			0.50 ***		
NT culms	0.44 ***	0.36 *					0.50 ***		
NT spikes	0.61 ***					0.36 *	0.61 ***		0.43 **
NHI	0.42 ***	0.42 ***					0.45 ***	0.52 ***	
CPNup				0.27 *	0.38 ***			0.52 ***	
Plant height									
Days to anthesis									−0.28*

Ant, anthesis; CPAA, contribution of post-anthesis assimilation to grain-filling; CPNup, contribution of post-anthesis nitrogen uptake to total nitrogen uptake; DM, dry matter; DMT, dry matter translocation; DMTEff, dry matter translocation efficiency; HI, harvest index; GNC, grain nitrogen concentration; GNS, grain number per spike; GNup, grain nitrogen uptake; GY, grain yield; Mat, maturity; N, nitrogen; NC, nitrogen concentration; NHI, nitrogen harvest index; NT, nitrogen translocation; NTEff, N translocation efficiency; NUE, N use efficiency; NutEff, N utilization efficiency; PAA, post-anthesis assimilation; PANup, post-anthesis nitrogen uptake; TKW, thousand kernel weight. Numbers of included genotypes are as indicated in **Table 2**. See **Supplementary Table 3** for correlations with grain protein deviation.

### Correlations With Grain Yield

In all years, GY correlated well (*p* < 0.001) with total DM at maturity (from r = 0.75*** in 2016 to 0.90*** in 2015), but with the HI only in 2016 (r = 0.56***), the year when HI was lowest (**Table 3**). For 2017, a trade-off between HI and the total DM is visible from the flattening regression line (**Figure 2A**). Despite medium correlations of total Ant DM with GY, the resulting DMT never correlated with GY, owing to the non-significant or even slightly negative correlations of the DMT efficiency. Thus, PAA explained GY better, from r = 0.42*** in 2016, to r = 0.71*** in 2015. N uptake at maturity, representing the N uptake efficiency under constant N fertilization, explained more variation in GY (r = 0.82***, 0.57***, 0.61***) than the N utilization efficiency for grain yield (NutEff_grain; r = 0.53***, 0.59**, n.s. in 2015, 2016, and 2017, respectively). In contrast to grain number per m² (**Supplementary Figure 1**, r = 0.56***, 0.33**, 0.49**), none of the three “direct” yield components correlated with GY in more than 1 year (**Table 3**). GY correlated (*p* < 0.001) with total Nup and GNup at maturity in all years but mostly not with Nup in vegetative organs. The only weak negative correlations between GY and GNC (–0.40***, –0.45***, and –0.43*) resulted in a substantial variation in GNup (**Figure 2B**).

### Correlations With Grain N Concentration

Besides GY, direct DM traits were not useful for describing GNC (**Table 3**). Total Nup and GNup exhibited low correlations with GNC in 2016 and 2017. PANup and its contribution to the total Nup (CPNup) exhibited weak positive correlations in 2015 and 2016, whereas robust negative correlations were observed of GNC with NutEff_total and especially NutEff_grain (–0.92***, –0.81***, –0.92***).

### Correlations With Grain N Uptake

Correlation patterns with GNup were similar as with grain yield (GY), especially in 2015 (**Table 3**). GNup correlated with GY particularly in the first year (r = 0.86***, 0.66***, and 0.64*** in 2015, 2016, and 2017, respectively), but only weakly with GNC in 2016 and 2017. A negative tendency was found with leaf N concentration at maturity. The total and organ-level NT and NT efficiency exhibited positive correlations (*p* < 0.001) in 2015. Conversely, total and spike NT positively but NT efficiency negatively NT correlated positively but NT efficiency negatively with GNup in 2017. In addition, post-Ant N uptake gave medium, positive correlations (r = 0.36–0.54) with GNup in all years. The NHI exhibited weak positive correlations in 2015 and 2016, compared with the dominant effect of the total Nup (r = 0.97***, 0.93***, and 0.95 ***; **Figure 2D**). Like for the DM-HI with the total DM, the NHI tended to decline for higher levels of total Nup in 2017 (**Figure 2D**). Fewer traits were significantly (p < 0.05) correlated with GPD (**Supplementary Table 3**), including in all years stronger correlations with GNC (r > 0.89) than with GNup (r = 0.51, 0.65, 0.76), but always negative correlations with NutEff_grain and NutEff_total. In both 2015 and 2016, PANup showed moderate correlations (r = 0.49) whereas total NT (r = 0.49) and culm NT (r = 0.46) only in 2017. Only in 2017, Ant traits, including total Nup, Nup of culms and spikes, and spike NC, were indicative for GPD, in contrast to correlations with total Nup at maturity (r = 0.51–0.73) in all years.

## Discussion

### Effects of Growing Conditions on Trait Characteristics and Stability

Significant genotypic effects and genotype × environment interactions were identified for all investigated traits (**Supplementary Table 2**). Maturity traits were often more heritable than Ant traits. Heritability (H²) was similarly high as that reported under French conditions in a historical cultivar set (Cormier et al., 2013) for the three yield components and plant height, slightly lower for GNC and GY, but higher than that reported in the American Great Plains (Guttieri et al., 2017). As in both studies, GY was more heritable than GNup. Adverse sowing conditions, along with low soil N supply and drought/heat effects during late grain filling in the first year accounted for low GY in 2015. In 2017, the pre-crop grass-clover, along with overall favorable growing conditions, increased GY to 9.9 t ha^–1^, which is beyond the average of 7.1 t ha^–1^ (at 0% moisture) reached on the regional farm level. GNup was lower than the fertilized N amount in 2015 (on average only 74%) and 2016 (82%) but far higher in 2017 (175%), being associated with the higher soil N supply for GNup in 3 years from 20, 44, to 111 kg ha^–1^, respectively, in the non-fertilized control plots.

### Relationships of Dry Matter Traits With Grain Yield

The results confirm the significance of “kernels per m²” (Shearman et al., 2005; Peltonen-Sainio et al., 2007; Reynolds et al., 2009; Slafer et al., 2014; Lynch et al., 2017) and the varying importance of the single yield components (Cormier et al., 2013). As GY, its two additive temporal components, DMT and PAA, reflected the year effects with the lowest contribution of post-Ant assimilation (CPAA) in 2016. Conversely, high DMT in 2016 was mostly driven by the highest DM at Ant, whereas DMT efficiency (DMTEff) was lower than in 2015. Low irradiance and high plant densities in 2016 might have decreased PAA and favored DM losses (Austin et al., 1977). Hence, the difference approach is likely to overestimate DMT and underestimate PAA because of neglecting losses by respiration and leaf shedding, which were quantified to be approximately one quarter of the loss of Ant DM (Austin et al., 1977; Savin and Slafer 1991; Gebbing and Schnyder, 1999). After Ant, low irradiance (Demotes-Mainard and Jeuffroy, 2004) and pathogen pressure (Bancal et al., 2007) could have limited PAA. The negative correlation (r = –0.41) between Ant Nup and PAA in that year suggests that higher Nup did not increase ongoing assimilation. A lack of positive correlations of DMTEff and GY and the low DMTEff in 2017 despite the highest GY might suggest that pre-Ant DM reserves were not fully exploited because of a persisting sink-limitation during grain filling (Schnyder, 1993; Reynolds et al., 2009; Serrago et al., 2013). Conversely, the higher DMTEff in the heat/drought-affected year 2015 and the disease-affected year 2016 indicates that additional assimilates were mobilized under differing types of stress conditions (Ehdaie et al., 2006). Concerning maturity traits, GY was primarily a function of total DM, whereas correlations with vegetative organs were lower. The correlations of straw DM (not shown) were in the range of those from organs best related to GY (culm in 2015 and 2017). Remarkably, a substantial trade-off between total DM and HI was only found in 2017 (**Figure 2A**), suggesting sink limitation under the conditions of exceptionally high PAA. Although further selecting for HI was suggested, and the hypothesized limitation of 0.62 is not near to be approached in the present set of genotypes, the results confirm the variation in HI to be secondary behind total DM (Unkovich et al., 2010; Foulkes et al., 2011).

### Uptake Rather Than Utilization Efficiency as a Driver for NUE

Total Nup correlated closer with total DM (r > 0.77) than NutEff_total (**Supplementary Equation 1e**), suggesting that Nup but not its conversion efficiency into DM was the primary driver for variation in DM accumulation. The slightly dominant effect of NupEff is consistent with findings of Latshaw et al. (2016) and Guttieri et al. (2017), whereas Le Gouis et al. (2000) and Gaju et al. (2011) reported NutEff to be more important, especially under high N supply. Differing conclusions in the literature are likely related to the different germplasms used. Notably, the low NutEff_grain in 2017 and its lacking correlation with GY only in this year seemed to be not only an effect of decreasing photosynthetic efficiency under the conditions of high N uptake, as NutEff_total did not decrease with total Nup in this year (not shown). Instead, the HI decreased with total Nup (r = –0.57) unlike in previous years, thereby partly counteracting the effect of Nup on GY. This underpins that GY was sink-limited in this year as also evidenced by the low DMTEff (Fischer, 2007; Parry et al., 2011; Gaju et al., 2011). Even if total and grain NutEff were more heritable across years than total Nup (0.50), they appear not suitable as indirect selection traits because of their complicated determination.

### Strategies for Increasing Grain N Uptake

NT dominated the N uptake into the grain (GNup) over post-Ant N uptake (PANup). In 2016, a substantial post-Ant loss in N was observed in some genotypes, whereas others still exhibited positive Nup up to 52 kg N ha^–1^. N loss by >60 kg ha^–1^ was noted under unfavorable conditions (Papakosta and Gagianas, 1991; Delogu et al., 1998; Guttieri et al., 2017; Ehdaie and Waines, 2001). Possibly, N was released by the straw but partly lost during the transport because of diseases, so that the loss was not captured in the apparent NT efficiency but accounted as an apparent net negative PANup in 2016 (Simpson and Dalling, 1981). Despite the lower contribution, post-Ant N uptake always correlated with GNup and with GPD in 2015 and 2016, in contrast to NT and NT efficiency, confirming previous results on its importance for GNup and GPD (Monaghan et al., 2001; Bancal et al., 2008; Bogard et al., 2010; Guttieri et al., 2015). Moreover, post-Ant N uptake confirmed its negative correlation with total Ant Nup (−0.33**, –0.72***, and –0.43* in the 3 years, respectively; not shown; Monaghan et al., 2001; Bogard et al., 2010), and with the resulting NT, due to limited N resources (Guttieri et al., 2017; Noulas et al., 2018). More clearly than total DM affected GY, total Nup dominated the variation in GNup between genotypes and years (**Figure 2**), whereas the NHI correlated only moderately with GNup in 2015 and 2016. The NHI was higher and similar as for American (Guttieri et al., 2017) and European (Cormier et al., 2013) genotypes, respectively, and moderately heritable. It was always positively correlated with the (apparent) NT efficiency (r = 0.70–0.85; not shown), as demonstrated previously (Hirel et al., 2007; Fageria, 2014). Unlike in previous years, in 2017, no positive correlation between the NHI and GNup, and a negative correlation between the NHI and total Nup, were observed (**Figure 2D**); thus, genotypes high in Nup might have encountered sink limitation not only for DM but also for GNup (Mi et al., 2000). In 2015 and 2016, the NHI correlated with the HI (r > 0.53), NutEff_grain, and further PAA and GY suggesting generally positive effects of the post-Ant metabolism both on the DM and N balance (Desai and Bhatia, 1978; Ehdaie and Waines, 2001; Guttieri et al., 2017). The inverse correlation between GY and GNC was weak compared with previous studies (Monaghan et al., 2001; Oury and Godin, 2007; Bogard et al., 2010; Guttieri et al., 2015; Latshaw et al., 2016; Thorwarth et al., 2018). Perhaps, a population of less selected breeding lines exhibits a weaker trade-off between GY and GNC than cultivars that are either optimized toward GY or GNC, thereby exhibiting more variation in GNup. In addition, more negative relationships may be found in more N-deficient conditions (Triboi et al., 2006). GNup as a product of GY and GNC was however always closer related to GY than to GNC, as reported previously (Heitholt et al., 1990; Le Gouis et al., 2000), suggesting that the selection for GY rather than GNC favors N uptake efficiency. In contrast, with the GNC∼GY regression being inclined from the isoline (**Figure 2B**), the selection for GPD would have favored genotypes high in GNC but not in GNup (Rapp et al., 2018). While Nup at Ant was associated with GY in all years, this was the case for GNup in 2015 and 2017, but for GNC and GPD only in 2017.

### Optimizing Grain N Concentration

Despite the positive correlation between GNC and total Nup, the markedly negative association with grain N utilization efficiency (NutEff_grain) and its both components NutEff_total and HI (**Table 3**; **Supplementary Equation 1g**) explains the GY/GNC antagonisms. In addition, the extraction of NHI from this equation illustrates that GNC theoretically increases and decreases with the NHI (Heitholt et al., 1990; Triboi et al., 2006) and the HI (Kramer, 1979; **Supplementary Equation 1h**), respectively, which, however, was only observable for the HI in 2016. Furthermore, the NHI/HI ratio exhibited a positive correlation in 2015 and 2017 (r = 0.28* and 0.59***); thus, NutEff_total (**Supplementary Equation 1h**) remained more explanatory for GNC (**Supplementary Figure 2**). Post-Ant N uptake (PANup) was better correlated both with GNC and GPD than NT, suggesting a reduced NutEff_total of the N taken up during the influence of senescence as confirmed by its negative correlation with PANup in 2015 and 2016 (not shown). This is in line with the reported association of PANup and GPD (Bogard et al., 2010).

## Conclusions

Different concepts investigated in this study regarding the variation in GY, GNup and GNC suggest the following conclusions: (i) Selection for GY using yield components was confirmed to be not promising due to only moderate correlations from grain number per m² only. (ii) Among Ant traits, both total DM and Nup correlated moderately with GY and with GNup but were not consistently exceeded for explaining GY by organ-level DM and Nup traits neither by Ant NC traits. (iii) Despite being a major source for GY, DMT and its efficiency were not predictive for GY, in contrast to PAA, which was also more heritable. (iv) In spite of being dominated by NT, variation in PANup appears to be equally important for maximizing GNup. (v) These results suggest that, under temperate conditions with favorable post-Ant conditions, the plant Ant status is not sufficient for predicting final GY and GNup, whereas stay-green traits should be considered as well due to the dominant role of the grain-filling phase. (vi) For maximizing total DM, the efficiency in N uptake was found to be more crucial than the internal utilization efficiency for DM. (vii) At maturity, total DM and total Nup largely dominated the variation in GY and GNup over the grain partitioning (i.e., harvest indices), respectively. (viii) The weak negative correlation between GNC and GY resulted into substantial variation in GNup. While the selection for GY would mostly also select genotypes superior in GNup, few stable correlations were found between GNC and directly determinable traits. (ix) Given that in most applied concepts, the accumulation of both DM and Nup was more descriptive than N concentration and the internal efficiencies of partitioning, translocation, and conversion, targeting phenotyping techniques more toward accumulative traits, i.e., direct DM and Nup is suggested—complemented through multi-temporal phenotyping for capturing traits during maturation.

## Data Availability

The raw data supporting the conclusions of this manuscript will be made available by the authors, without undue reservation, to any qualified researcher.

## Author Contributions

YH, LP and US conceived and designed the experiments; LP performed the experiments; LP analyzed the data; LP and US wrote the paper.

## Funding

This research was funded by the DFG (German Research Foundation)-funded project SCHM 1456/6-1.

## Conflict of Interest

The authors declare that the research was conducted in the absence of any commercial or financial relationships that could be construed as a potential conflict of interest.
