# Supplementary figures and images for: Temporal Dynamics and the Contribution of Plant Organs in a Phenotypically Diverse Population of High-Yielding Winter Wheat: Evaluating Concepts for Disentangling Yield Formation and Nitrogen Use Efficiency
Source: Front Plant Sci. 2019 Oct 29;10:1295. doi: 10.3389/fpls.2019.01295 (PMC6829449; doi:10.3389/fpls.2019.01295)

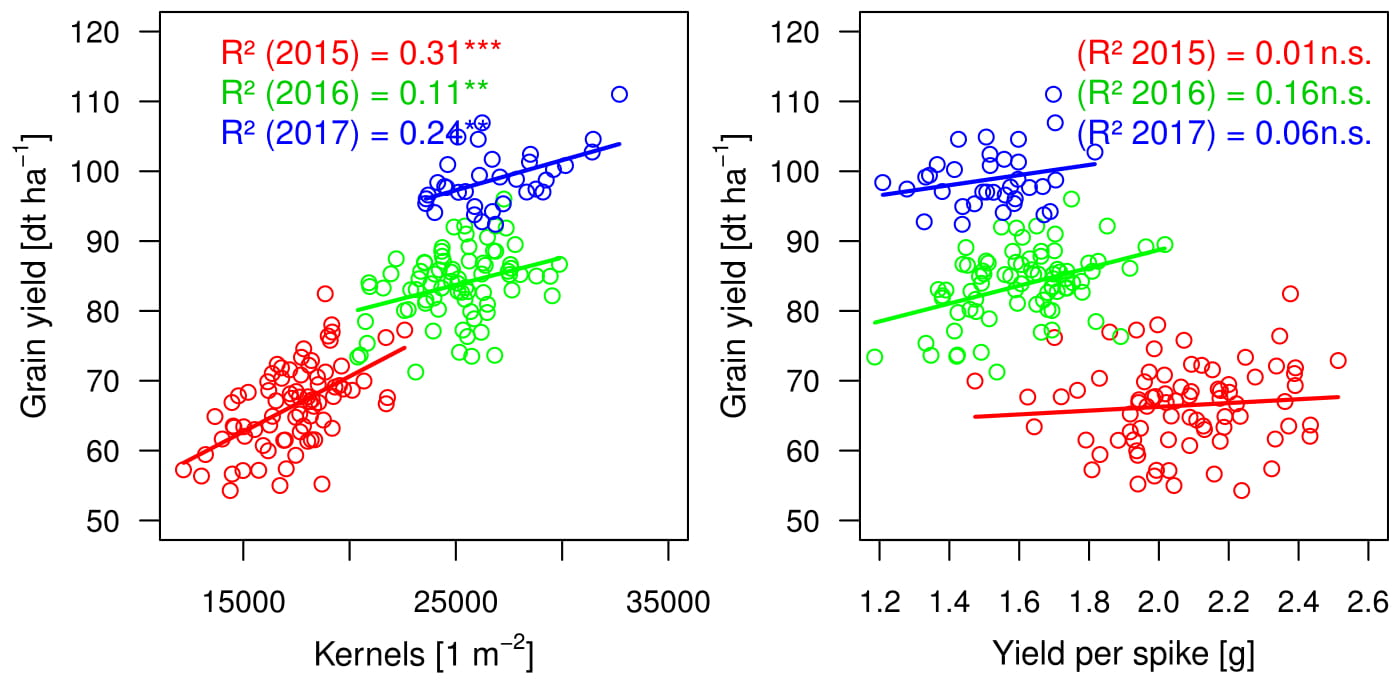

Supplement: Supplementary file 1 [file Image_1.jpeg]

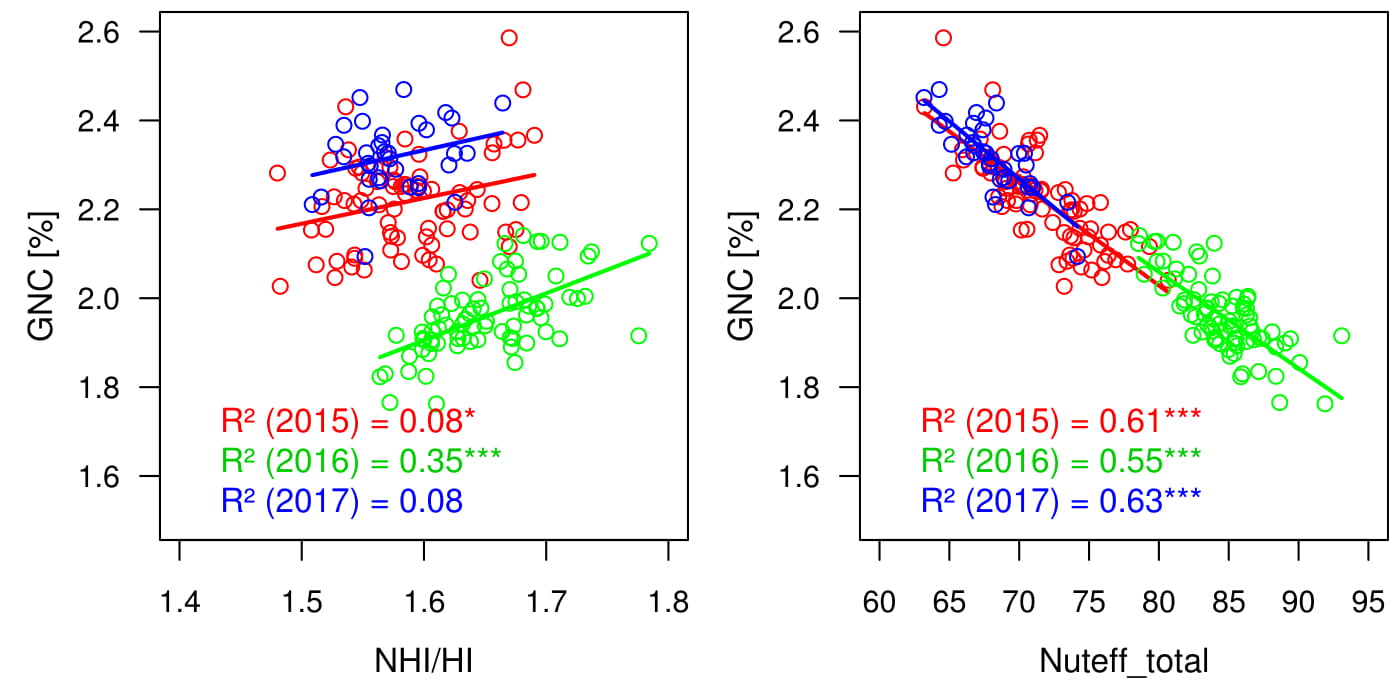

Supplement: Supplementary file 2 [file Image_2.jpeg]
