# Supplementary material for: Temporal Dynamics and the Contribution of Plant Organs in a Phenotypically Diverse Population of High-Yielding Winter Wheat: Evaluating Concepts for Disentangling Yield Formation and Nitrogen Use Efficiency
Source: Front Plant Sci. 2019 Oct 29;10:1295. doi: 10.3389/fpls.2019.01295 (PMC6829449; doi:10.3389/fpls.2019.01295)
Supplement: Supplementary file 3 [file Table_1.docx]

Supplementary Material

# Supplementary Figures and Tables

## Supplementary Figures


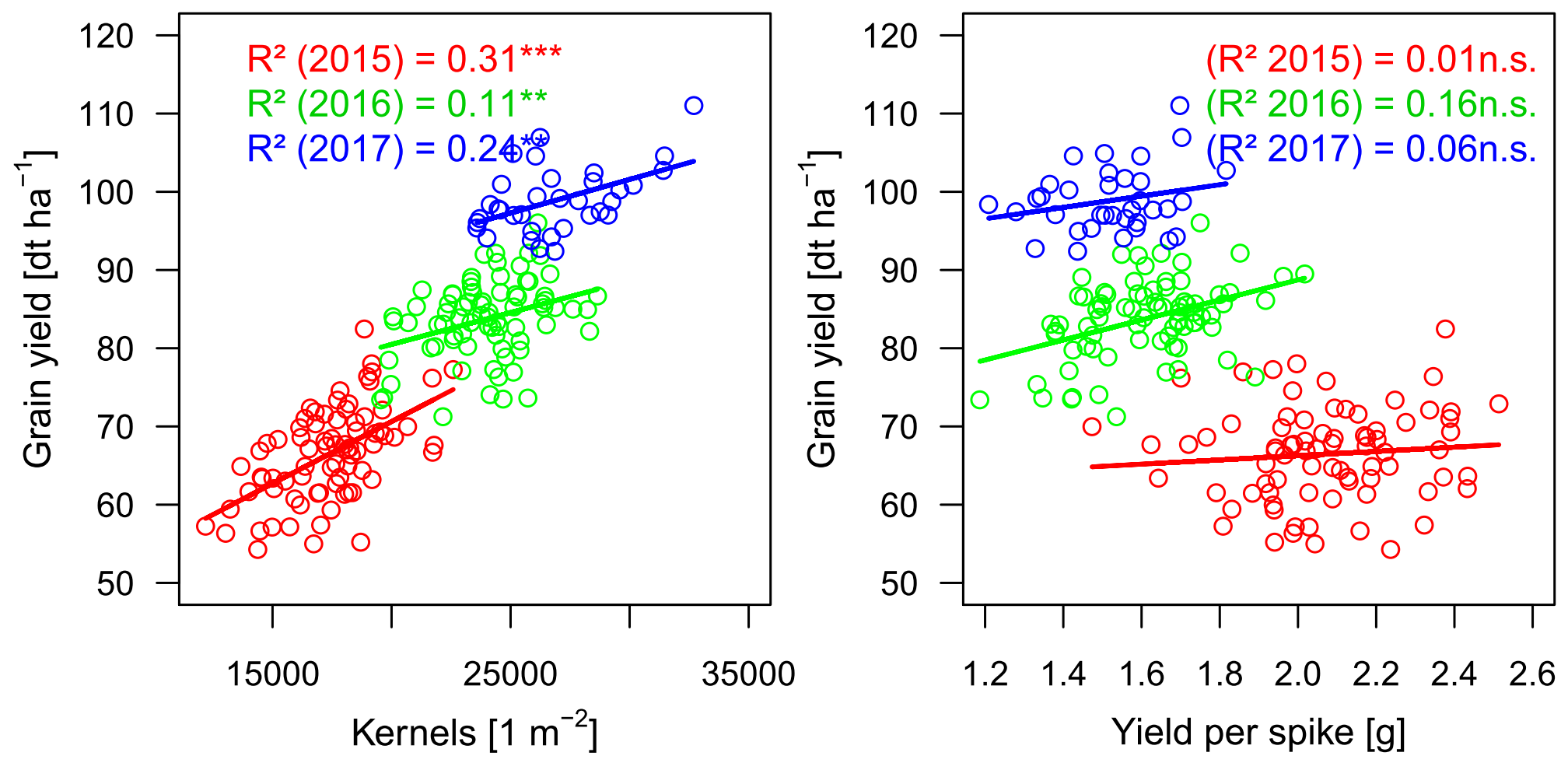


Supplementary Figure 1: Aggregated grain yield components and relationship with grain yield: Left: The relationship between grain yield and grain number per area. Right: The relationship between grain yield and yield per spike.


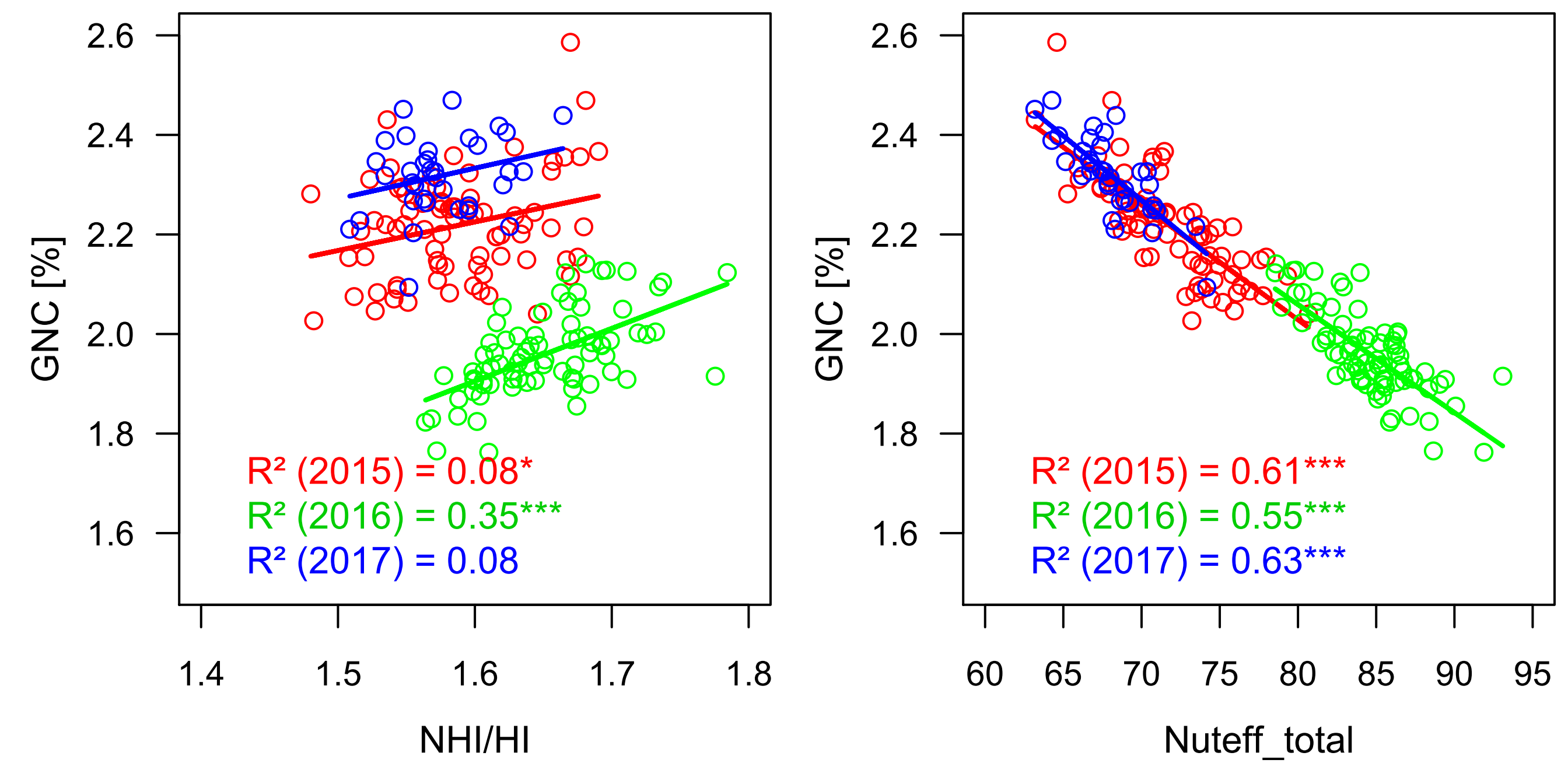


Supplementary Figure 2: Illustration of Supplementary Equation 1: Grain N concentration (GNC) related to the ratio of N harvest index (NHI) and harvest index (HI; left) and to the total N utilization efficiency (right).

## Supplementary Tables

Supplementary Table 1: List of traits considered: Direct dry matter (DM) and N uptake (Nup) traits, derived DM and N traits and N concentration (NC) traits.

| **Trait group** | **Trait name** | **Abbreviation** |
| --- | --- | --- |
| Dry matter (DM) | total DM at anthesis | DM Ant |
|  | total DM at maturity | DM Mat |
|  | grain DM at maturity | DM grain Mat |
|  | leaves DM at anthesis | DM leaves Ant |
|  | culms DM at anthesis | DM culms Ant |
|  | leaves DM at maturity | DM leaves Mat |
|  | culms DM at maturity | DM culms Mat |
|  | chaff DM at maturity | DM chaff Mat |
| derived DM traits | spike density | spike density |
|  | contribution of pre-anthesis assimilation to grain filling | CPreAA |
|  | post-anthesis assimilation | PAA |
|  | DM translocation | DMT |
|  | DM translocation efficiency | DMTEff |
|  | harvest index | HI |
|  | grain nitrogen utilization efficiency | NutEff_grain |
|  | total nitrogen utilization efficiency | NutEff_total |
|  | grain number per spike | GNS |
|  | thousand kernel weight | TKW |
| N concentration (NC) | spikes nitrogen concentration at anthesis | NC spikes Ant |
|  | leaves nitrogen concentration at anthesis | NC leaves Ant |
|  | culms nitrogen concentration at anthesis | NC culms Ant |
|  | leaves nitrogen concentration at maturity | NC leaves Mat |
|  | culms nitrogen concentration at maturity | NC culms Mat |
|  | grain nitrogen concentration at maturity | NC grain Mat |
|  | chaff nitrogen concentration at maturity | NC chaff Mat |
| N uptake (Nup) | post-anthesis nitrogen uptake | PANup |
|  | spikes nitrogen uptake at anthesis | Nup spikes Ant |
|  | leaves nitrogen uptake at anthesis | Nup leaves Ant |
|  | culms nitrogen uptake at anthesis | Nup culms Ant |
|  | total nitrogen uptake at anthesis | Nup Ant |
|  | leaves nitrogen uptake at maturity | Nup leaves Mat |
|  | culms nitrogen uptake at maturity | Nup culms Mat |
|  | grain nitrogen uptake at maturity | Nup grain Mat |
|  | total nitrogen uptake at maturity | Nup Mat |
|  | chaff nitrogen uptake at maturity | Nup chaff Mat |
|  | straw nitrogen uptake at maturity | Nup straw Mat |
| derived N traits + yield components | contribution of post-anthesis N uptake to total nitrogen uptake | CPNUp |
|  | nitrogen harvest index | NHI |
|  | spikes nitrogen translocation | NT spikes |
|  | leaves nitrogen translocation | NT leaves |
|  | culms nitrogen translocation | NT culms |
|  | total nitrogen translocation | NT |
|  | nitrogen translocation efficiency | NTEff |
| other traits | height milk ripeness |  |
|  | days to anthesis in June |  |

Supplementary Table 2: ANOVA F-values for genotype (G) effect within years and G, environment (E) an G*E interaction effects calculated from a mixed model across years with fixed E and G effects and random effects for replicate, row and column. Number of genotypes (n) area as listed in Table 2. Maximum, mean, minimum and standard deviation are given and colored (low-high) for each trait. If not indicated, traits are unitless. Abbreviations are as follows: Ant, anthesis; Mat, maturity; GY, grain yield; GNC, grain nitrogen concentration; GNup, grain nitrogen uptake; DM, dry matter; N, nitrogen; NC, nitrogen concentration; DMTEff, dry matter translocation efficiency; PAA, post-anthesis assimilation; DMT, dry matter translocation; HI, harvest index; TKW, thousand kernel weight; GNS, grain number per spike; CPAA, contribution of post-anthesis assimilation to grain-filling; NutEff, N utilization efficiency; NTEff, N translocation efficiency; PANup, post-anthesis nitrogen uptake; NT, nitrogen translocation; NHI, nitrogen harvest index; CPNup, contribution of post-anthesis nitrogen uptake to total nitrogen uptake.

|  |  | **F-value within years** | | | **F-value across years** | | | **maximum** | | | **mean** | | | **minimum** | | | **St. deviation** | | |
| --- | --- | --- | --- | --- | --- | --- | --- | --- | --- | --- | --- | --- | --- | --- | --- | --- | --- | --- | --- |
| **Trait  group** | **trait** | **`15** | **`16** | **`17** | **E** | **G** | **G*E** | **`15** | **`16** | **`17** | **`15** | **`16** | **`17** | **`15** | **`16** | **`17** | **`15** | **`16** | **`17** |
| **DM traits [kg ha^-1^]** | **DM Ant** | 2.1* | 2.4*** | 3.8*** | 157.79*** | 2.16*** | 1.76*** | 10755 | 16001 | 13863 | 8245 | 13303 | 12085 | 5951 | 11312 | 10220 | 915 | 857 | 725 |
|  | **DM leaves Ant** | 1.8 | 3.5** | 6.2*** | 74.82*** | 3.48*** | 1.99*** | 1933 | 2916 | 3095 | 1430 | 2381 | 2638 | 923 | 2014 | 2189 | 195 | 222 | 217 |
|  | **DM culms Ant** | 2.3* | 3.8*** | 4.7*** | 148.79*** | 2.46*** | 2.77*** | 6954 | 9768 | 8130 | 5211 | 8166 | 6819 | 3693 | 7002 | 5720 | 601 | 646 | 464 |
|  | **DM spikes Ant** | 2.9** | 2.4* | 4.7*** | 155.58*** | 3.32*** | 1.75*** | 1980 | 2765 | 3065 | 1604 | 2330 | 2627 | 1118 | 1883 | 2145 | 203 | 181 | 191 |
|  | **DM Mat** | 2.2* | 5.1*** | 6.9*** | 101.5*** | 5.27*** | 1.98*** | 15395 | 20819 | 21246 | 12544 | 18005 | 18948 | 9627 | 15895 | 17186 | 1132 | 949 | 932 |
|  | **DM leaves Mat** | 1.8 | 15.4*** | 5.8*** | 72.97*** | 5.74*** | 1.83*** | 1287 | 1639 | 2030 | 932 | 1292 | 1575 | 644 | 910 | 1180 | 119 | 153 | 163 |
|  | **DM culms Mat** | 2.9** | 17.9*** | 6*** | 94.57*** | 6.73*** | 2.06*** | 4527 | 7081 | 6795 | 3539 | 6066 | 5168 | 2563 | 4944 | 4093 | 405 | 494 | 451 |
|  | **DM chaff Mat** | 2* | 7.3*** | 3.8*** | 112.23*** | 3.6*** | 2.23*** | 2166 | 2479 | 2718 | 1386 | 2102 | 2297 | 738 | 1755 | 1936 | 227 | 160 | 169 |
|  | **DM grain Mat** | 2.9** | 8.8*** | 9.7*** | 106*** | 7.16*** | 2.8*** | 8246 | 9603 | 11103 | 6687 | 8380 | 9908 | 5428 | 7125 | 9240 | 596 | 496 | 368 |
| **N concentration traits [%]** | **NC leaves Ant** | 2* | 4.3*** | 5.2*** | 174.14*** | 2.96*** | 3.1*** | 3.08 | 3.46 | 3.83 | 2.72 | 3.00 | 3.51 | 2.29 | 2.53 | 2.91 | 0.17 | 0.20 | 0.16 |
|  | **NC culms Ant** | 4.5*** | 17.5*** | 4.8*** | 122.62*** | 3.08*** | 5*** | 0.96 | 1.24 | 1.31 | 0.82 | 1.04 | 1.17 | 0.69 | 0.88 | 1.06 | 0.06 | 0.09 | 0.06 |
|  | **NC spikes Ant** | 3.6** | 14.2*** | 9.9*** | 68.81*** | 6.78*** | 3.98*** | 1.87 | 1.90 | 2.10 | 1.70 | 1.75 | 1.91 | 1.53 | 1.55 | 1.70 | 0.07 | 0.08 | 0.09 |
|  | **NC leaves Mat** | 8.2*** | 27.1*** | 5.5*** | 88.97*** | 7.98*** | 11*** | 1.30 | 1.98 | 0.97 | 0.93 | 1.20 | 0.80 | 0.68 | 0.84 | 0.61 | 0.12 | 0.23 | 0.07 |
|  | **NC culms Mat** | 7.4*** | 3.3** | 4.1*** | 54.45*** | 4.27*** | 3.24*** | 0.45 | 0.38 | 0.53 | 0.29 | 0.34 | 0.45 | 0.23 | 0.28 | 0.35 | 0.04 | 0.02 | 0.04 |
|  | **NC chaff Mat** | 3.8*** | 7.6*** | 2.7*** | 7.33* | 4.84*** | 1.66** | 0.80 | 0.64 | 0.69 | 0.56 | 0.53 | 0.58 | 0.42 | 0.43 | 0.49 | 0.08 | 0.06 | 0.04 |
|  | **NC grain Mat** | 5.5*** | 8.8*** | 8*** | 168.85*** | 10.46*** | 2.7*** | 2.59 | 2.14 | 2.47 | 2.20 | 1.96 | 2.32 | 2.03 | 1.76 | 2.09 | 0.10 | 0.08 | 0.07 |
| **N uptake traits [kg ha^-1^]** | **Nup Ant comb** | 1.6 | 2.7*** | 2.9*** | 153.57*** | 1.76*** | 1.74*** | 136 | 253 | 251 | 109 | 207 | 223 | 76 | 176 | 191 | 12.6 | 13.5 | 14.4 |
|  | **Nup leaves Ant** | 1.6 | 3.5** | 4.1*** | 102.14*** | 2.41*** | 2.59*** | 55 | 87 | 111 | 39 | 72 | 92 | 24 | 59 | 66 | 6.2 | 8.3 | 8.6 |
|  | **Nup culms Ant** | 1.9 | 3.9*** | 3.1*** | 82.27*** | 1.49* | 2.85*** | 55 | 99 | 93 | 42 | 85 | 80 | 32 | 72 | 66 | 4.9 | 7.6 | 6.6 |
|  | **Nup spikes Ant** | 2.9** | 2.1* | 4.7*** | 168.45*** | 3.05*** | 1.9*** | 36 | 49 | 62 | 27 | 41 | 50 | 19 | 32 | 40 | 3.4 | 3.2 | 4.8 |
|  | **Nup Mat** | 1.3 | 3.2*** | 5.2*** | 89.13*** | 2.59*** | 1.71*** | 210 | 247 | 311 | 173 | 213 | 278 | 146 | 189 | 252 | 13.2 | 10.9 | 13.5 |
|  | **Nup leaves Mat** | 1.8 | 10.9*** | 4.5*** | 26.77*** | 2.96*** | 3.57*** | 14 | 20 | 16 | 9 | 15 | 13 | 6 | 12 | 8 | 1.4 | 2.3 | 1.8 |
|  | **Nup culms Mat** | 2* | 4.3*** | 4.8*** | 36.65*** | 2.96*** | 2.49*** | 16 | 26 | 33 | 10 | 21 | 23 | 8 | 16 | 17 | 1.4 | 2.0 | 3.1 |
|  | **Nup chaff Mat** | 2.6** | 5.9*** | 3.9*** | 44.13*** | 3.06*** | 1.8*** | 11 | 15 | 18 | 8 | 11 | 13 | 5 | 8 | 10 | 1.5 | 1.5 | 1.6 |
|  | **Nup straw Mat** | 1.4 | 3.6*** | 5*** | 46.5*** | 3.12*** | 1.7*** | 35 | 62 | 67 | 27 | 49 | 49 | 20 | 40 | 37 | 3.1 | 4.1 | 5.6 |
|  | **Nup grain Mat** | 1.7 | 4.7*** | 5.6*** | 131.81*** | 3.52*** | 2.08*** | 185 | 193 | 253 | 147 | 164 | 229 | 122 | 142 | 213 | 12.3 | 9.8 | 9.4 |
| **derived DM traits + yield components** | **DMTEff** | 2.7** | 3.4*** | 2.5*** | 5.22* | 2.63*** | 1.93*** | 0.38 | 0.39 | 0.38 | 0.29 | 0.27 | 0.25 | 0.17 | 0.11 | 0.19 | 0.04 | 0.05 | 0.04 |
|  | **PAA [kg ha^-1^]** | 3.5** | 3.3*** | 3.7*** | 67.89*** | 2.75*** | 1.88*** | 6407 | 8021 | 8631 | 4302 | 4693 | 6864 | 2870 | 1874 | 4737 | 699 | 944 | 675 |
|  | **DMT [kg ha^-1^]** | 2.3* | 2.7*** | 2.4*** | 79.47*** | 1.69*** | 1.74*** | 3747 | 6235 | 4800 | 2385 | 3701 | 3045 | 1384 | 1297 | 2062 | 500 | 796 | 533 |
|  | **HI** | 5.2*** | 5.9*** | 4.7*** | 328.05*** | 7.63*** | 2.13*** | 0.59 | 0.50 | 0.55 | 0.53 | 0.47 | 0.52 | 0.49 | 0.43 | 0.47 | 0.02 | 0.02 | 0.01 |
|  | **TKW [g]** | 21*** | 16.4*** | 23*** | 48.17*** | 27.65*** | 5.45*** | 47 | 42 | 46 | 39 | 35 | 37 | 30 | 29 | 33 | 4.0 | 2.9 | 2.7 |
|  | **spike density [m^-2^]** | 3.3** | 5.9*** | 9.2*** | 227.25*** | 7.7*** | 1.74*** | 476 | 633 | 814 | 326 | 524 | 659 | 247 | 407 | 559 | 44 | 47 | 52 |
|  | **GNS** | 17.5*** | 8.3*** | 22.3*** | 171.73*** | 17.6*** | 2.23*** | 72 | 61 | 55 | 54 | 46 | 41 | 38 | 32 | 30 | 6.9 | 5.1 | 4.4 |
|  | **CPAA** | 3** | 3.2*** | 2.6*** | 29.65*** | 2.19*** | 1.99*** | 0.8 | 0.97 | 0.78 | 0.64 | 0.56 | 0.69 | 0.47 | 0.23 | 0.5 | 0.07 | 0.10 | 0.06 |
|  | **NutEff_total** | 3.1** | 5.1*** | 4.0*** | 354.34*** | 6.48*** | 1.94*** | 81 | 93 | 74 | 73 | 85 | 68 | 63 | 79 | 63 | 3.5 | 2.9 | 2.4 |
|  | **NutEff_grain** | 6.3*** | 6.5*** | 4.9*** | 32.34*** | 7.6*** | 2.68*** | 42 | 43 | 39 | 39 | 39 | 36 | 32 | 34 | 32 | 1.9 | 2.0 | 1.4 |
| **derived N traits** | **NTEff** | 5.9*** | 4.4*** | 2.5*** | 7.95* | 3.59*** | 2.89*** | 0.80 | 0.81 | 0.81 | 0.75 | 0.76 | 0.78 | 0.66 | 0.71 | 0.73 | 0.03 | 0.02 | 0.02 |
|  | **PANup [kg ha^-1^]** | 2.6* | 4*** | 1.3*** | 258.56*** | 2.11*** | 2.15*** | 89 | 52 | 82 | 65 | 5 | 56 | 44 | -58 | 37 | 10.1 | 15.3 | 9.9 |
|  | **NT [kg ha^-1^]** | 2.2* | 3*** | 1.9*** | 224.63*** | 1.66*** | 1.9*** | 105 | 201 | 196 | 82 | 159 | 173 | 56 | 126 | 152 | 11.0 | 12.9 | 10.9 |
|  | **NT leaves [kg ha^-1^]** | 2* | 4.4*** | 3.6*** | 132.84*** | 2.41*** | 3.09*** | 44 | 74 | 95 | 30 | 56 | 80 | 16 | 41 | 53 | 5.6 | 8.5 | 7.9 |
|  | **NT culms [kg ha^-1^]** | 2.2* | 5.3*** | 2*** | 127.93*** | 1.56** | 3.37*** | 43 | 81 | 67 | 32 | 64 | 57 | 21 | 49 | 47 | 4.3 | 8.1 | 5.2 |
|  | **NT spikes [kg ha^-1^]** | 4.1*** | 1.9 | 3.9*** | 232.01*** | 2.36*** | 2.01*** | 27 | 35 | 46 | 19 | 29 | 37 | 12 | 20 | 28 | 2.8 | 3.0 | 4.0 |
|  | **NHI** | 4.4*** | 7.6*** | 5.4*** | 205.17*** | 6.55*** | 2.86*** | 0.88 | 0.81 | 0.86 | 0.85 | 0.77 | 0.82 | 0.79 | 0.72 | 0.79 | 0.02 | 0.02 | 0.01 |
|  | **CPNup** | 2.9** | 1.9* | 1.2*** | 388.68*** | 2.36*** | 2.49*** | 0.48 | 0.21 | 0.27 | 0.37 | 0.02 | 0.20 | 0.27 | -0.30 | 0.13 | 0.05 | 0.04 | 0.03 |
|  | **Plant height [m]** | 6.75*** | 18.70*** | 4.7*** | 1575.9*** | 1.74*** | 0.84 | 0.75 | 1.07 | 0.73 | 0.61 | 0.93 | 0.65 | 0.49 | 0.80 | 0.55 | 0.05 | 0.05 | 0.05 |
|  | **Days to anthesis [d]** | - | - | - | 355.86*** | 69.09*** | 9.52*** | 16.00 | 18.00 | 19.00 | 12.85 | 10.61 | 12.87 | 11.00 | 2.00 | 5.00 | 1.80 | 2.77 | 2.63 |

## Supplementary Equation

Supplementary Equation 1: Extension of Equation 1: Relating NUE_grain to GY and Nup and the contributing components.

(a)$\boldsymbol{NUE}_{\boldsymbol{grain}}\mathbf{=}\boldsymbol{NupEff}\mathbf{*}\boldsymbol{NutEf}\boldsymbol{f}_{\boldsymbol{grain}}$

NutEff_grain can be expressed as the product of NutEff_total and HI:

(b) $\boldsymbol{NUE}_{\boldsymbol{grain}}\mathbf{=}\boldsymbol{NupEff}\mathbf{*}\boldsymbol{NutEf}\boldsymbol{f}_{\boldsymbol{total}}\mathbf{*}\boldsymbol{HI}$

With dividing NUE and NupEff by available or fertilized nitrogen, GY becomes a function of total Nup and NutEff_grain:

(c) $\boldsymbol{GY}\mathbf{=}\boldsymbol{Nup}_{\boldsymbol{total}}\mathbf{*}\boldsymbol{NutEf}\boldsymbol{f}_{\boldsymbol{grain}}$

(d) $\boldsymbol{GY}\mathbf{=}\boldsymbol{Nup}_{\boldsymbol{total}}\mathbf{*}\boldsymbol{NutEf}\boldsymbol{f}_{\boldsymbol{total}}\mathbf{*}\boldsymbol{HI}$

Eliminating HI expresses total DM as function of Nup and NutEff_total.

(e) $\boldsymbol{DM}_{\boldsymbol{total}}\mathbf{=}\boldsymbol{Nup}_{\boldsymbol{total}}\mathbf{*}\boldsymbol{NutEf}\boldsymbol{f}_{\boldsymbol{total}}$

Replacing GY from (d) by GNup/GNC:

(f) $\frac{\boldsymbol{GNup}}{\boldsymbol{GNC}}\mathbf{=}\boldsymbol{Nup}_{\boldsymbol{total}}\mathbf{*}\boldsymbol{NutEf}\boldsymbol{f}_{\boldsymbol{total}}\mathbf{*}\boldsymbol{HI}$

Resolving for GNC:

(g) $\frac{\boldsymbol{GNup}}{\boldsymbol{Nup}_{\boldsymbol{total}}\mathbf{*}\boldsymbol{NutEf}\boldsymbol{f}_{\boldsymbol{total}}\mathbf{*}\boldsymbol{HI}}\mathbf{=}\boldsymbol{GNC}$

Extracting NHI:

(h) $\boldsymbol{NHI}\mathbf{*}\frac{\mathbf{1}}{\boldsymbol{NutEf}\boldsymbol{f}_{\boldsymbol{total}}\mathbf{*}\boldsymbol{HI}}\mathbf{=}\boldsymbol{GNC}$
