# Supplementary material for: Temporal Dynamics and the Contribution of Plant Organs in a Phenotypically Diverse Population of High-Yielding Winter Wheat: Evaluating Concepts for Disentangling Yield Formation and Nitrogen Use Efficiency
Source: Front Plant Sci. 2019 Oct 29;10:1295. doi: 10.3389/fpls.2019.01295 (PMC6829449; doi:10.3389/fpls.2019.01295)
Supplement: Supplementary file 4 [file Table_2.docx]

Supplementary Table 3: Correlations (only p < 0.05) with grain protein deviation (GPD), by traits grouped as in Table 2. Bold numbers highlight correlations among the three target traits. See Supplementary Table 1 for a description of all traits. Numbers of included genotypes are as indicated in Table 2.

|  | 2015 | **2016** | **2017** |
| --- | --- | --- | --- |
| DM Ant |  |  | 0.44 ** |
| DM leaves Ant |  |  | 0.41 * |
| DM culms Ant |  |  | 0.42 * |
| DM spikes Ant |  |  |  |
| DM Mat |  |  |  |
| DM leaves Mat |  |  | 0.49 ** |
| DM culms Mat |  |  |  |
| DM chaff Mat |  |  |  |
| DM grain Mat (GY) |  |  |  |
| NC leaves Ant |  |  |  |
| NC culms Ant |  |  |  |
| NC spikes Ant |  |  | 0.4 * |
| NC leaves Mat |  |  |  |
| NC culms Mat |  |  |  |
| NC chaff Mat |  | **0.34 *** | **0.45 **** |
| NC grain Mat (GNC) | 0.92 *** | 0.89 *** | 0.9 *** |
| total Nup Ant |  |  | 0.56 *** |
| Nup leaves Ant |  |  |  |
| Nup culms Ant |  |  | 0.54 *** |
| Nup spikes Ant |  |  | 0.37 * |
| total Nup Mat | 0.51 *** | 0.6 *** | 0.73 *** |
| Nup leaves Mat |  |  | 0.55 *** |
| Nup culms Mat | 0.29 ** | 0.45 ** | 0.37 * |
| Nup chaff Mat |  | 0.43 * | 0.33 * |
| Nup straw Mat |  |  | 0.49 ** |
| Nup grain Mat (GNup) | 0.51 *** | 0.65 *** | 0.76 *** |
| DMTEff |  | -0.23 * |  |
| PAA |  |  |  |
| DMT |  | -0.22 * |  |
| HI |  |  | -0.36 * |
| TKW | 0.26 * |  |  |
| spike density |  |  |  |
| GNS | -0.33 ** | -0.24 * |  |
| CPAA |  |  |  |
| NutEff_total | -0.68 *** | -0.69 *** | -0.66 *** |
| NutEff_grain | -0.77 *** | -0.61 *** | -0.88 *** |
| NTEff |  |  |  |
| PANup | 0.49 *** | 0.49 *** |  |
| NT |  |  | 0.49 ** |
| NT leaves |  |  |  |
| NT culms |  |  | 0.46 ** |
| NT spikes | 0.25 * |  |  |
| NHI |  | 0.36 ** |  |
| CPNup | 0.25 * | 0.49 *** |  |
| Plant height |  |  |  |
| Days to anthesis |  |  |  |
